# Using a Commercially Available App for the Self-Management of Hypertension: Acceptance and Usability Study in Saudi Arabia

**DOI:** 10.2196/24177

**Published:** 2021-02-09

**Authors:** Tourkiah Alessa, Mark S Hawley, Nouf Alsulamy, Luc de Witte

**Affiliations:** 1 Centre for Assistive Technology and Connected Healthcare School of Health and Related Research University of Sheffield Sheffield United Kingdom; 2 Biomedical Technology Department College of Applied Medical Sciences King Saud University Riyadh Saudi Arabia; 3 Public Health School of Health and Related Research The University of Sheffield Sheffield United Kingdom; 4 College of Business University of Jeddah Jeddah Saudi Arabia

**Keywords:** mHealth, mobile phone, hypertension, usability, acceptance, user satisfaction

## Abstract

**Background:**

The use of smartphone apps to assist in the self-management of hypertension is becoming increasingly common, but few commercially available apps have the potential to be effective along with adequate security and privacy measures in place. In a previous study, we identified 5 apps that are potentially effective and safe, and based on the preferences of doctors and patients, one (Cora Health) was selected as the most suitable app for use in a Saudi context. However, there is currently no evidence of its usability and acceptance among potential users. Indeed, there has been little research into the usability and acceptance of hypertension apps in general, and less research considers this in the Gulf Region.

**Objective:**

This study aims to evaluate the acceptance and usability of the selected app in the Saudi context.

**Methods:**

This study used a mixed methods approach with 2 studies: a usability test involving patients in a controlled setting performing predefined tasks and a real-world usability study where patients used the app for 4 weeks. In the usability test, participants were asked to think aloud while performing the tasks, and an observer recorded the number of tasks they completed. At the end of the real-world pilot study, participants were interviewed, and the mHealth App Usability Questionnaire was completed. Descriptive statistics were used to analyze quantitative data, and thematic analysis was used to analyze qualitative data.

**Results:**

In total, 10 patients completed study 1. The study found that app usability was moderate and that participants needed some familiarization time before they could use the app proficiently. Some usability issues were revealed, related to app accessibility and navigation, and a few tasks remained uncompleted by most people. A total of 20 patients completed study 2, with a mean age of 51.6 (SD 11.7) years. Study 2 found that the app was generally acceptable and easy to use, with some similar usability issues identified. Participants stressed the importance of practice and training to use it more easily and proficiently. Participants had a good engagement level with 48% retention at the end of study 2, with most participants’ engagement being classed as meaningful. The most recorded data were blood pressure, followed by stress and medication, and the most accessed feature was viewing graphs of data trends.

**Conclusions:**

This study shows that a commercially available app can be usable and acceptable in the self-management of hypertension but also found a considerable number of possibilities for improvement, which needs to be considered in future app development. The results show that there is potential for a commercially available app to be used in large-scale studies of hypertension self-management if suggestions for improvements are addressed.

## Introduction

### Background

Hypertension is one of the most common chronic diseases in adults and can lead to several serious complications, including stroke, heart disease, and renal failure. The condition affects approximately one billion people globally. In Saudi Arabia, 27.2% of people aged above 30 years have been diagnosed with hypertension [1-5]. Lowering blood pressure (BP) lessens the risk of complications, but many patients with hypertension do not control their BP well [3,5,6]. Self-management is one of the most effective ways to control hypertension. This involves encouraging patients to take control of their condition, for instance, by changing their lifestyle, by becoming more involved in their treatment, and by managing their symptoms and psychosocial and physical effects [7-9]. However, self-management behavior remains to be difficult and is an aspect of treatment with which patients often struggle.

The increase in smartphone use in recent years has led to an increase in health-related apps on these devices. In Saudi Arabia alone, there were an estimated 21.8 million smartphone users in 2018, and as a result, the use of health apps as a means for treating patients has increased [10,11]. Many commercial apps are available, offering a potential way to promote and assist the self-management of hypertension [12-15].

This study focuses on one commercially available app (Cora Health) developed by Swiftware. This app was selected based on the findings of previous studies. Alessa et al [16] conducted a systematic review of apps intended to assist in the self-management of hypertension and found that these are potentially effective in lowering BP, particularly when they have comprehensive functionalities, including self-monitoring, reminders, and educational information or automatic feedback. Most apps were developed specifically for an individual study, and there is still a lack of research evidence supporting the effectiveness and usability of commercially available apps. A recent review of apps actually available in app stores found that only few apps (30/186, 16.1%) had the potential to be effective, very few apps (5/186, 2.6%) had the potential to be effective and with adequate security and privacy safeguard, and none of them claim to have involved users in their development [15]. A subsequent study explored patients’ and doctors’ preferences toward the 5 apps found to be effective and with adequate security and privacy measures. When participants were asked to rate the apps, the Cora Health app was considered the most suitable. However, there is still no published evidence regarding its usability or effectiveness [15].

Many commercial apps, including the Cora Health app, did not involve users in app development, although many studies have found that participants’ acceptance of apps and their perceptions of usefulness and ease of use are all key factors for mobile health (mHealth) adoption [17-19]. Moreover, there is very little research into usability and acceptance in general [15,16] and even less research that specifically examines the Saudi context or the wider Gulf Region. However, Clemmensen et al [20] suggested that usability problems could be influenced by users’ cultures, experience, and knowledge. This highlights the importance of assessing a commercially available app’s usability, acceptance, and engagement before its effectiveness can be evaluated [21-23]. This study aims to assess the usability and acceptance of the Cora Health app to support the self-management of hypertension in Saudi Arabia, which is the first study in this context. The objectives of this study are as follows: (1) to assess how usable the app is; (2) to assess patients’ experience using the app: what barriers to use they see and whether they think it could be improved; and (3) to examine how participants engage with the app. Our central hypothesis was that users would find the app acceptable and usable overall but that they might also identify usability issues or specific preferences and needs, which the app did not meet. 

### Study Design and Methods

This research used a convergent mixed methods approach to comprehensively assess the app’s usability and acceptance [24], conducted via 2 studies: (1) a usability test and (2) a real-world usability and acceptance study.

The qualitative and quantitative data in this research were collected concurrently in both studies and analyzed separately. It was then integrated and synthesized in the interpretation so that the facets of the results could be examined together and compared. Efficient integration of both qualitative and quantitative methods results in a larger knowledge yield than that obtained by treating the 2 strands in isolation [24,25].

Usability is defined as, “the extent to which a product can be used by specific users to achieve specific goals with effectiveness, efficiency and satisfaction in a specific context of use” [26]. Acceptance, for the purpose of the study, includes participants’ actual app use, their satisfaction, and attitudes toward using the app and intention or willingness to continue using the app [27].

### Participants

The population of this study included Saudi adults with hypertension. Participants for both studies were purposively selected from 1 hospital and 2 primary care centers in Riyadh, Saudi Arabia, based on the predefined inclusion and exclusion criteria [28].

For the usability test, a sample size of 10 eligible participants was used. This is sufficient to discover more than 80% of usability problems [29,30]. For the usability and acceptance study, the target sample size was 20 to 30 eligible people, which is a similar number to previous studies assessing the acceptance and usability of health apps [17,31,32].

The inclusion criteria in both studies were as follows: at least 30 years old; diagnosed with hypertension (stages 1-3) as a primary disease for a minimum of 6 months; able to speak Arabic, give consent, and actively participate in the study; and possess or have access to an iOS-compatible smartphone. The exclusion criteria were as follows: having a cognitive impairment that limits the ability to give informed consent or to actively take part in the studies; having prehypertension or hypertension during pregnancy; being unable to read and understand the Arabic language; and (for study 2 only) affected by stage 4 or severe hypertension (≥180/110 mm Hg).

### Participant Recruitment

The study was conducted at the largest hospital related to the Ministry of Health and 2 primary care centers related to the hospital. Participants in study 1 were recruited via posters and flyers advertising the study, with recruitment continuing until data saturation was reached, that is, once new participants were no longer revealing new data, information, or usability issues. [33]. For study 2, physicians were approached to recruit participants from among their patients based on the study’s eligibility criteria. People who expressed an interest in participating in the studies were provided with a further information sheet and a consent form.

### Intervention

**Figure 1:**
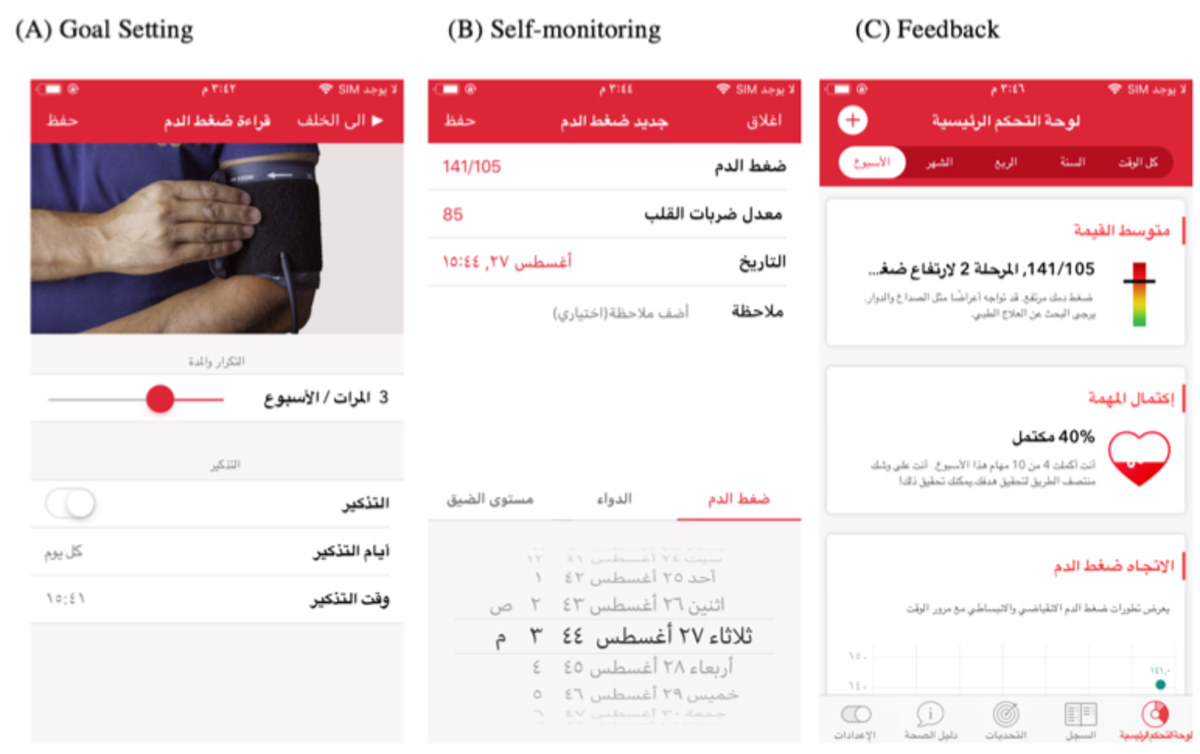
The main functionalities of the app.

Figure 1 shows the app version used runs on the iPhone. The app was translated into Arabic by the researcher and then back into English to check for translation accuracy. Samples of the Arabic version were then sent to a test group of Arabic speakers with hypertension to check its comprehensibility and clarity. Owing to developer constraints, it was not possible to translate the complete app content; some small sections, for example, labels of figures and names of medication, remained in English.

The app has 3 main features: monitoring (BP, stress, and medication), setting weekly challenges, and medical information. The BP feature allows users to upload their BP measurements to the device either automatically or manually, displays the readings on graphs, and includes feedback. This app also allows users to enter their distress level and its reasons as well as medication names and doses. The second feature allows users to set weekly challenges, such as monitoring BP. The app also has educational materials that allow patients to learn how to self-manage their chronic conditions.

## Methods

### Study 1: Usability Testing

The usability of the app was studied using a thinking-out-loud technique, where participants verbalize their thoughts and feelings while using the app and performing a set of predefined tasks. An observer collected first impressions and initial reactions [21]. A pilot of the usability study was undertaken with 2 eligible participants before the commencement of the full study.

The tasks presented to the participants were based on the main functionalities of the app, ensuring that the app was fully tested and used. The tests were audiorecorded to aid analysis. Participants were given multiple attempts to complete the tasks. If the participant was unable to complete a task after several attempts, assistance was offered. Each session lasted approximately 40-60 min and was conducted by the researcher with a facilitator aiding observations and taking notes.

Each session began by briefly introducing the test aim and its procedures and explaining the think-aloud technique and the purpose of the app. The participants were asked to sign a consent form and complete a short questionnaire, including demographic questions and smartphone experience. The participants then performed the tasks and vocalized their reactions. The observer recorded the number of tasks participants completed, any requests for assistance, and errors made. The observer also asked questions during the tasks to encourage participants to share their opinions. Finally, the participants were given an opportunity to raise any issues relating to topics that were not covered.

### Study 2: Real-World Usability and Acceptance Study

A one-group posttest study was carried out to analyze how the app was used in everyday life as a part of the participants’ routines. This study assessed the acceptance and usability of the app by means of a questionnaire, user engagement data, and a post interview after 4 weeks of using the app. Owing to the study aims and methods, patients and investigators were not blinded.

Each participant was asked to sign a consent form and complete a brief demographic questionnaire, including smartphone experience. The app was then downloaded onto the patient’s iPhone. Face-to-face training was provided by the researcher, and the instruction manual was provided. Participants were provided with a validated home Omron M7 BP monitoring device [34-36]. Participants could obtain technical support throughout the study from the researcher by email or phone. For quality and safety reasons, patients continued their usual treatment with their physician. At the 4-week follow-up, participants completed the usability questionnaire and were interviewed using a semistructured interview to assess their personal experience, including acceptance of using the app and their views on its usability. The interviews lasted approximately 40 min and were audiorecorded, and concurrent notes were taken. Finally, the BP devices were collected by the researcher.

#### User Engagement Data

Information on how often participants used the app was automatically (anonymously) recorded. Participants were supplied with a specific link to download the Arabic version created for the study. The engagement data were provided anonymously, where the app did not collect data on a per-user basis due to data privacy regulations. We recorded the number of log-ins, the types and frequencies of data entered, and the number and frequency of features accessed. These are the 3 most common measurements used to assess user engagement with health apps [37]. The study also examined the user’s session duration and user engagement over time.

#### Usability Questionnaire

The mHealth App Usability Questionnaire (MAUQ) was used [38]. The questionnaire was translated into Arabic following the guidance offered by the World Health Organization [39]. The pilot study found this translated questionnaire to have a Cronbach α of .9, a scale level content validity index of 0.98.

### Data Analysis

SPSS software (package 19) [40] was used to calculate the descriptive statistics for the quantitative analysis. All qualitative data were transcribed, checked for accuracy, and analyzed using thematic analysis [40,41]. The qualitative analysis followed 6 steps: (1) data familiarization, (2) creation of initial codes, (3) collection of codes into broader themes, (4) specification of themes, (5) review of themes, and (6) writing the report [41].

The thematic analysis was partly deductive and partly inductive. In total, 2 researchers (TA and NA) independently analyzed 20% of transcripts. The researchers then checked for consensus on these coding. This resulted in standardized codes, in which TA was used for the remaining transcripts. Any new codes were added when necessary.

On the basis of the study aims, the initial themes were devised deductively. Additional themes and subthemes were then devised inductively based on users’ initial expectations and their experiences of the app. Final themes and subthemes were confirmed through discussion among the authors. Following data analysis, an integration matrix [24,25] was used to compare data from the different methods. The quantitative and qualitative results of the research were integrated and analyzed together, considering any convergences and divergences between these different data. The matrix is given in Multimedia Appendix 1.

## Results

### Study 1: Usability Testing

#### Participant Characteristics

The usability study was completed by 10 participants, aged 35 to 69 years, with a mean of 48.8 (SD 11.7) years. In total, 6 participants were female and 4 were male. Overall, 6 participants had a diploma degree (a level of Saudi qualification between high school and bachelor’s degree) or higher. Most participants (9/10, 90%) had experience using smartphones for longer than 3 years. Most participants (8/10, 80%) had hypertension for 1 year or more (Table 1).

**Table 1:** Characteristics of usability test participants.

Characteristics		Values
**Age (years)**		
	Mean (SD)	48.8 (11.7)
	Range	35-69
**Age groups (years), n (%)**		
	30-39	2 (20)
	40-49	3 (30)
	50-59	2 (20)
	≥60	3 (30)
**Gender, n (%)**		
	Male	4 (40)
	Female	6 (60)
**Time since diagnosed with hypertension (years), n (%)**		
	<1	2 (20)
	1-3	4 (40)
	>3	4 (40)
**Education level**		
	Less than high school	3 (30)
	High school	1 (10)
	Diploma	2 (20)
	Undergraduate degree	2 (20)
	Postgraduate degree	2 (20)
**Smartphone users**		
	Yes	10 (100)
	No	0 (10)

#### Usability Test Results

The analysis of the usability test transcripts resulted in 2 themes: overall usability and user satisfaction and app content to support self-management.

#### Overall Usability and User Satisfaction

Users gave numerous positive comments relating to the usability of the Cora Health app interface and were generally satisfied. They described it as *helpful*, *fab*, and *easy to use*, and some asked to continue using the app by downloading the original English version from the app store. Patients felt that using the app would help improve their understanding of hypertension and their management of the condition. More than half of the participants completed all but 2 tasks. Participants often needed assistance while performing tasks, as they were unfamiliar with the app. They would require some time to become familiar with the app before being able to use it proficiently. The theme of overall usability and user satisfaction is separated into the 2 subthemes of app accessibility and user interface issues. Further details of these are presented in Multimedia Appendix 2.

#### App Content to Support Self-Management

User comments on the app content were generally positive, with participants describing it as “useful in self-managing.” Some users liked the information available in the health guide and accompanying the BP feedback. They felt that this would increase their understanding and encourage them to take action to control their BP:

I really love the additional explanation. It offers some helpful advice that encourages me to take action because my BP is not normal. It helps me to understand my situation and to do something to control [my BP].

P2

Some participants expressed that the tick feature of the app (ticking off completed tasks) would encourage completion of the challenges:

it encourages me to do more tasks.

P4

#### Task Completion

Most participants downloaded the app (task 1) without any assistance, except for 3 older participants who required help. However, this difficulty may have been related to the method of downloading the trial version, which is different from a typical app. All participants completed both the *Entering Stress Data* task (task 3.2), inserting a tick to mark self-monitoring as completed (task 8), and *indicate how many challenges* are set (task 9), without making any errors or asking for any assistance. Therefore, these tasks had the highest completion rate.

Very few users completed task 7: setting a reminder for self-monitoring BP. Only 20% (2/10) of participants completed this task without errors, whereas 80% (8/10) of participants completed the task with errors. Similarly, in task 10, only 2 participants completed the task without errors, whereas 5 participants (5/10, 50%) completed the task with errors and 3 participants (3/10, 30%) required help.

The remaining tasks were completed by most participants without errors or assistance (60% for tasks 2, 3.1, 4, and 11; 70% for tasks 5 and 6). The full completion, error, and assistance rates are presented in Table 2.

**Table 2:** Usability test tasks.

Task		Participants who completed the task, n	Participants who made errors, n	Participants who needed an assistance, n
1. Downloading the app and log-in		7	0	3
2. Monitoring and registering BP^a^ with the app		6	0	4
**3. Recording other data (** **medication and distress)**				
	3.1 Enter the medication name and its dose	6	4	0
	3.2 Enter the distress level you feel and select any applicable problems	10	0	0
4. Indicate whether the BP average value and see if its normal or not		6	2	2
5. Compare the new measurement of BP data with those measured before		7	1	2
6. Indicate whether the current measured BP is normal or not using the Blood Pressure Scatter Char		7	1	2
7. Set a reminder for self-monitor BP 4 times a week		2	8	0
8. Inserting a tick to mark self-monitoring as completed		10	0	0
9. Indicate how many challenges are set		10	0	0
10. Indicate how many tasks have you completed and how many tasks do you still to need finish		2	5	3
11. Read the information about BP monitors		6	0	4

^a^BP: blood pressure.

### Study 2: Real-World Usability and Acceptance Study

#### Participant Characteristics

In total, 23 participants agreed to participate in this study. A total of 2 participants decided to withdraw after a few days because of their busy schedule. One other participant had to withdraw because of technical issues related to their device. In total, 20 participants (11 males and 9 females) completed the study. They were aged between 33 and 71 years, with a mean of 51.6 (SD 11.7) years. Overall, 80% (16/20) of participants had a diploma degree or higher. Most participants had experience using smartphones for more than 3 years. Most participants (16/20, 80%) had hypertension for 1 year or more (Table 3).

**Table 3:** Participant characteristics.

Characteristics		Values
**Age (years)**		
	Mean (SD)	51.6 (11.7)
	Range	33-71
**Age groups (years), n (%)**		
	30-39	3 (15)
	40-49	6 (30)
	50-59	6 (30)
	≥60	5 (25)
**Gender, n (%)**		
	Male	11 (55)
	Female	9 (45)
**Time since diagnosed with hypertension (years), n (%)**		
	<1	4 (20)
	1-3	7 (35)
	>3	9 (45)
**Education level, n (%)**		
	Less than high school	4 (20)
	High school	0 (0)
	Diploma	5 (20)
	Undergraduate degree	7 (35)
	Postgraduate degree	4 (20)
**Smartphone users, n (%)**		
	Yes	20 (100)
	No	0 (0)

#### Usability Questionnaire

This section presents the results pertaining to the usability of the app and participants’ satisfaction (as measured by the MAUQ). Participants perceived the app as a useful tool (mean score 6.3, SD 0.40 on a scale of 1-7). They were also satisfied with the app and its interface (mean score 6.2, SD 0.25) and expressed that the app was easy to use (mean score 6, SD 0.2).

Participants scored high when asked whether the app is a useful tool in helping them to manage their condition effectively (mean score 6.6, SD 0.50). A high score was also given when asked whether the app is useful for receiving health care services such as accessing educational information, tracking their own activities, and performing self-assessment (mean score 6.7, SD 0.47). However, participants scored lower (mean score 5.7, SD 1.49) when asked if they could use the app even when the internet connection was poor or unavailable.

#### App Engagement Data

Table 4 shows group-level data on participants’ engagement with the app over a month, measured by the length of time of each user’s session. The average session duration was 1 min and 35 seconds, with around 72.9% (346/474) of users’ sessions being in the *meaningful engagement* ranges of 30 to 60 seconds or longer [42].

**Figure 2:**
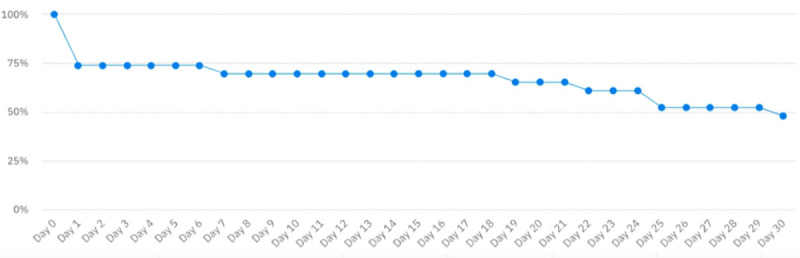
Participant engagement over time.

Figure 2 shows the retention data for study participants, that is, the number of users who continued to use the app. A total of 6 users ceased using the app following the first day’s use. From day 1 to day 6, 74% of the participants were active. From day 7 to 18, 70% were active. User retention then gradually decreased to 48% on day 30.

On average, the app was opened 21.4 times per user, totaling 493 times over a month, as shown in Table 5. The most accessed functionality was viewing the Logbook, which allows users to self-monitor their previously entered data. The least accessed functionality was setting behavior goals (*Challenge Created*).

**Table 4:** Participant session duration.

Sessions durations	Sessions, n
0 seconds	0
0-3 seconds	38
3-10 seconds	40
10-30 seconds	50
30-60 seconds	96
1-30 minute	141
3-10 minute	93
10-30 minute	16

**Table 5:** App functionalities use.

App section and app functionalities			Participants who used the functionalities, n		Times functionality was used, n		Average per participant
**General**							
	App opened	23		493		21.4	
	App closed	23		768		33.4	
**Logbook and dashboard**							
	Feedback on behavior or outcome of behavior (dashboard viewed)	23		1002		43.6	
	Self-monitoring of previous data (logbook viewed)	23		1384		60.2	
	Self-monitoring of blood pressure	21		416		19.8	
	Self-monitoring of medication	20		234		11.7	
	Self-monitoring of distress	20		246		12.3	
**Challenges**							
	Setting behavior goal	19		72		3.78	
	Review of behavior goal(s)	21		704		33.52	
	Task completed	19		721		37.94	

The most common self-monitoring behavior was entering BP, followed by stress and medication: each user self-monitored their BP and inputted an average of 19.8 times.

The user engagement data over the month for entering BP, medication, and stress levels and for viewing previously entered data are presented in Multimedia Appendix 3. These graphs clearly show a distinction between a group of users who engage regularly (more than 6 times) and a group with lower engagement figures. The majority of participants engaged regularly with the app. In total, 15 (71.4%) participants recorded data for at least 3 days per week (the level considered sufficient for treatment and adherence with self-monitoring [43]). The figures for recording emotion and medication use are lower.

The user engagement data over the month for goal setting (challenges), task completion for each goal, and reviewing behavior goals are presented in Multimedia Appendix 3. Most participants (n=19) set at least one goal, with a number ranging from 1 to 10. Most participants (n=14) set 3 or more different types of goals.

#### Interview Results

Analysis of interviews resulted in 3 themes: usage of the app, capacity to support self-management, and usability of the app. Some participants’ quotations for this section are provided in Multimedia Appendix 4.

#### Usage of the App

##### General Satisfaction and Use

Patients were satisfied with the app and saw it as an *enjoyable*, *interesting, accessible and convenient, useful,* *and informative* tool in managing hypertension. They generally found it easy to incorporate the app into their routines. Few participants (n=2) felt that the app felt the app required time and effort.

Patients expressed that they would be likely to continue using this app after the study and that they would recommend it to others. They gave several reasons, including that the app offered a single easily accessible location for their data, that it provides a good overview of their BP level, and because of its ease of use and convenience for managing their diseases.

##### App Functionality Use

Participants used most of the app functionalities. Interviewees reported that the most commonly used app function was self-monitoring BP data, followed by viewing the graphs and lists showing trends over a week or month. They stated that the visual representations were valuable, as was the quick and direct transmission of BP data from a BP monitoring device to the app.

Most people recorded medications and stress and perceived the facility to enter these as a positive feature. They also reported setting different types of challenges, the most common being entering BP and taking medication. Most also set challenges for other activities intended to increase exercise, and a few set challenges to reduce stress.

Hypertension information was generally considered useful, but a few patients stated that information was lacking, or already known, for example, regarding meals (especially Saudi foods), mental health, smoking, or information about pregnancy. Some participants emphasized that it was the overall range of functionalities that made the app valuable and worth using.

##### External Factors Influencing Use of the App

Participants reported a range of external factors that affected their use of the app. Family was one of the key factors mentioned, as both a motivating and demotivating factor for using the app. Busy lifestyles and other health issues reportedly prevented some participants from undertaking and/or completing additional challenges.

#### Capacity to Support Self-Management

##### A Daily Monitoring Tool

The app provides patients with a routine and structured system, helping them to maintain discipline in self-monitoring different types of data. Transmission of BP data by Bluetooth was easier and quicker than conventional recording methods or relying on memory. Presenting data immediately in a graphical view helped patients see trends over time.

Monitoring emotions and indicating the reasons behind them was a positive feature, but it would be good to allow monitoring of the symptoms that patients feel.

##### An Informative Tool

Participants found the feedback functionalities to provide a clear picture of BP levels and to show the relationship between their challenges completed and their BP level. However, participants would have liked more detail in the feedback on task completion and more tailoring of BP feedback to their individual cases.

##### A Commitment Tool

Participants expressed that this app increased their commitment and encouraged them to add and achieve more self-management strategies to their routine. Some participants also found that repeated reminders further encouraged them to complete their activities, but showing more written details would encourage them more rather than relying on notification alone. In total, 2 participants felt that it would increase their commitment if they were able to set their own challenges.

##### A Communication Tool

Participants felt that the app would be a valuable tool to increase patients’ participation in their care, for example, by aiding communication and sharing of data at doctors’ visits more easily than carrying manual copies. This aspect of the app was considered particularly beneficial for assisting during medical emergencies, with one suggesting that doctors should have real-time access to the app data.

#### Usability of the App

##### Overall Usability

Most participants found the app easy to use and reported high levels of confidence. Those who had little experience required some practice to increase their confidence or ability, some being more reliant on the study training and instructions or assistance.

Most participants found it easy to navigate the app and enter data, except when attempting to enter multiple readings at once, which requires the save button to be pressed multiple times. The ability to enter data retrospectively and to edit previously entered data were valued features. However, patients reported some issues with the *tick* feature for marking completed challenges, either because of physical difficulties in using the functionality or being unable to undo *ticks* made in error. To improve this feature, some participants suggested that ticking could be prompted by the app.

##### App Accessibility

Some participants, particularly older people, had difficulty reading text within the app. They liked the zoom feature in the health guide but would like to be able to change the font size elsewhere. Some suggested that the contrast in the BP entry screen could be improved to make buttons easier to find.

Not all words in this version were translated into Arabic (eg, medication names), which was an issue for some participants, as was the default calendar (ie, Gregorian rather than Islamic).

#### Suggested Improvements

The study results found some aspects of the app that should be considered for improvement. There is a need to increase the app accessibility by (1) translating some words into Arabic (eg, medication names); (2) allowing changing between Islamic and Gregorian calendars; (3) increasing the color contrast in the BP entry screen; and (4) allowing changing font size. Suggested improvements regarding user interfaces include (1) using clear and meaningful terminology (such as health information); (2) making the data entry button more visible; (3) allowing inputting of multiple data at once; (4) adding text in some charts to supplement the color coding; and (5) displaying a message when tasks are marked as complete or making this data visible from the Challenge screen.”

There were also some more general recommendations regarding app content. The feedback of BP should be more detailed and tailored for individual cases. The app should allow entering the symptoms that patients feel and allow them to set their own challenges. The *tick* function could be improved if it prompted participants to tick off items they have set reminders for and if it allowed participants to edit a tick when it was made in error.

## Discussion

### Principal Findings

This study evaluated a commercially available app that was carefully selected on the basis of existing evidence regarding the effectiveness, usability, privacy and security, and preferences of end users and health care professionals. This process resulted in selecting an app that was expected to have the best potential for being usable, acceptable, and effective in general, particularly in the Saudi context. The results presented in this paper do indeed show relatively good usability outcomes but still a considerable number of possibilities for improvement because of users’ differing needs, expectations, and preferences that need to be considered [44]. The actual usage data show that even this *best practice* app is not used by all participants and that only some of the functionalities are regularly used. This demonstrates the complexity of *getting it right* when developing smartphone apps and once again emphasizes the importance of acceptability, usability, and effectiveness evaluation, among target users, who are rarely consulted or involved in the commercial app development process, typically only being asked to evaluate once the app is released [45].

The results suggest that the provision of training could be a possible way to mitigate most usability issues and enhance user acceptance. The interview and MAUQ showed that the app is easy to use and generally accepted by participants. Some of these participants commented that training and instructions helped them use it more easily. The usability test, however, found that app usability was only moderate, and participants needed some familiarization time before they could use the app proficiently. A small number of tasks remained uncompleted by most people. Similarly, in the interview and questionnaire, participants reported that it was easy to navigate the app and enter data, whereas the usability test showed that participants faced issues with these aspects. This difference might be a consequence of the usability test being conducted in a controlled setting, in which participants had not received any previous training or practice. This finding appears to be in line with previous evidence indicating that a wide range of different users can use apps given the right training and support [46-48].

The results indicate that commercial apps have the potential to be met with sustained engagement from users. Engagement data showed that users’ sessions with the app were similar to other studies using similar apps for other chronic diseases [42]. However, the actual usage data show a higher level of sustained engagement with the app, with a 48% retention rate on day 30, in comparison with another study of self-monitoring apps, which showed a retention of 3.3% [49]. This study also found much higher levels of BP monitoring than some other studies concerning other chronic conditions. Most participants (71.4%) recorded their BP around 3 times or more per week. In contrast, Goyal et al [44] found that only 9% of participants achieved similar levels of engagement (≥3 times). There are several possible explanations for this, with potential implications for future research and app development: doctors asked patients to record BP measurements twice for each reading to ensure accuracy, which could increase the frequency of measuring [43]. This study did not provide patients with a secondary phone, which could have led to higher engagement [44]; the app’s feature for transmitting data either automatically or manually could have increased BP measurement. User motivation has been shown to be key to adherence with self-monitoring [22], so this may be another factor.

The study showed that participants in all strands expressed enthusiasm for using an app to support self-management because of its benefits in increasing their understanding and participation. All strands found that the app content (eg, information, etc) was considered a good potential tool to support self-management and to increase participants’ understanding and commitment. However, participants’ responses in interviews also revealed several concerns or limitations, suggesting that an app alone would not be a sufficient tool for self-management. Some external factors and barriers, for example, family, affected participants’ use of the app such as positively or negatively affecting patients’ optimism and self-esteem, or easing the stress of using the app to support the self-management of their disease [50]. These factors must also be considered when assessing the benefits of using apps to support the self-management of hypertension.

The usability test and interviews reported similar difficulties with app accessibility, for example, font size and color scheme. Previous research has shown that older people are likely to encounter more difficulties and have lower engagement with these types of technological interventions [51,52]. It is therefore important to assess how engagement levels might differ between younger and older participants and who are most likely to benefit from these interventions, particularly because most patients with hypertension are older [52]. Owing to data privacy regulations, the engagement data were collected anonymously in this study, so it is not possible to compare older and younger participants. These older members of the study sample highlighted some issues regarding the accessibility of the app, such as the inability to change font sizes, app presentation, data entry, and the need for help from family members. This suggests that the engagement of these older members of the population might be improved if such accessibility issues were addressed. Understanding and considering older adults’ opinions and needs is crucial to help introduce apps to this population and maximize their usability [53].

Participants in this study suggested that sharing their data with health care professionals for ongoing care should be effectively supported. Previous studies have suggested that apps that share health data with health care professionals can aid in treatment, especially in emergencies [54]. Apps that are limited to one specific condition may be less helpful if not properly integrated with health information systems, particularly for patients who have comorbid conditions that might complicate their treatment needs and require a large treatment team [54,55]. However, there are several potential barriers to mHealth integration with existing systems that should be considered [56].

### Strength and Limitations

Our study has several strengths. First, it evaluates the selected app, Cora Health, in 2 different situations: in real-life and under controlled settings, integrating different methods (eg, interview, questionnaire, etc) to gain in-depth insight and provide a complete picture of the usability of the app. As the convergent and divergent results from these strands indicate, such a mixed methods approach yields a more detailed picture of the research area. Second, through our analysis, we were able to identify areas where usability was a potential concern. From these findings, we were able to comprehensively establish ways to further refine the app to make it more usable. These conclusions could be extended to other mHealth apps. Third, this is the first study to evaluate the usability and acceptance of a commercially available app for people with hypertension in Saudi Arabia.

However, there are also some limitations to this study. For instance, the study only focused on patients’ opinions without considering health care professionals’ or experts’ opinions, which might have provided different clinical insights. This is because the app did not support any access for health care professionals. The small number of participants and selection bias are likely to have been other limitations: in order to be eligible, participants had to own an iOS-compatible smartphone; and as recruitment for the usability test was conducted via posters and flyers, the sample was therefore self-selecting and may have been biased in favor of highly motivated individuals. For the interviews, participants were recruited via their physicians. The study also used self-selecting and purposive sampling, which may be influenced by errors in judgment or assumptions by the researcher, leading to higher levels of bias and lower reliability. The number of older participants in the study sample was relatively low (8/30, 27%). This may have been a limitation, especially because the majority of patients with hypertension are older people. For these reasons, the generalizability of the study to the general population is somewhat limited. Despite attempts by the researcher and moderator to create a comfortable and welcoming research space, it is possible that the presence of a session moderator in the usability test may have affected user confidence or performance in a way that they may have differed from field use. Similarly, the potential generalizability of these findings beyond the Saudi context may also be limited: it may only be possible to generalize them to similar cultural contexts and to health care settings that are similar to the Saudi Ministry of Health. Finally, this study showed engagement over a 30-day period. As such, it is not possible to draw conclusions as to whether this might be sustained over a longer period. The study was concerned with describing users’ engagement rather than examining how this engagement might contribute to achieving certain health outcomes or behavior change, meaning it is also not possible to draw conclusions as to whether this constituted *effective engagement*.

### Recommendation for Further Studies

On the basis of the study results, it is important for future studies to investigate whether the levels of engagement recorded in this study could be sustained over the longer term to achieve the desirable outcomes [48,57]. There is also a need to evaluate the effectiveness of the app as well as *effective engagement*, that is, engagement that achieves desired behavior changes [58,59] and compare these results with usual care to reach clinical conclusions. This would require studies with larger numbers of users and longer follow-up periods. Future research should also consider how participant age might influence their engagement and should also examine contexts outside Saudi Arabia. Some issues raised by participants in this study will need to be addressed before the Cora Health app can be maximally effective in large-scale studies. Future studies should undertake a more collaborative approach between app developers and potential users to be mutually beneficial and lead to higher quality apps that can more fully support patients’ self-management.

### Conclusions

This study showed that a commercially available app can be usable and acceptable for the self-management of hypertension. Participants were generally satisfied and found that the selected app was easy to use and useful in supporting their self-management activities. However, some participants experienced issues with the app’s interface that need to be considered in future studies and app development. The results of this study suggest that there is potential for a commercially available app to be used in large-scale studies of the self-management of hypertension if suggestions for improvements are addressed.

## Appendix

Multimedia Appendix 1 Data Integration matrix.

Multimedia Appendix 2 Usability test data.

Multimedia Appendix 3 Engagement data.

Multimedia Appendix 4 Qualitative data.

## Abbreviations

BP: blood pressure
MAUQ: mHealth App Usability Questionnaire
mHealth: mobile health
