# Supplementary material for: Using a Commercially Available App for the Self-Management of Hypertension: Acceptance and Usability Study in Saudi Arabia
Source: JMIR Mhealth Uhealth. 2021 Feb 9;9(2):e24177. doi: 10.2196/24177 (PMC7902196; doi:10.2196/24177)
Supplement: Multimedia Appendix 1 [file mhealth_v9i2e24177_app1.docx]

|  | **Usability Test**  *(QUAN+QUAL)* | **Engagement data**  *(QUAN)* | **Questionnaire**  *(QUAN)* | **Interview**  *(QUAL)* | **Comments to integration** |
| --- | --- | --- | --- | --- | --- |
| **Theme 1: Usability of the app** |  |  |  |  |  |
| **Overall usability** | App generally easy to use; more than half of participants (n=7) complete most tasks (8/10) but they stressed they need time until be proficient with the app,  Few tasks (setting reminder and found how many tasks completed) were uncompleted by most people. |  | Most participants agreed app easy to use (18/20) and easy to learn with a mean of (5.8), and all agreed that they recovered any mistake they made easily. | Most participants, who have little experience of technology found app easier than those who have little experience. They also commented provided training & instruction helped them to found it easy. | Somewhat (**Discordant**), interview and questionnaire are similar in founding app easy to use, but interview provide further details (**expansive**) that training, instruction and more practice helped to be easier. However, usability test showed that the app has good usability and they stressed they need time until be proficient with the app |
| **App accessibility** | Some aspects like small font size in some section, less contrast between background and button, requiring an internet connections and words have not translated into Arabic (e.g medication names) were faced as struggle to engage users with the app. |  |  | Concerns were raised about too small font size, non-preferred calendar, less contrast between background and button and untranslated English words into Arabic and suggested to improve them. | Both UT and interviews had **similar** concerns and difficulties and suggested to change font size, change to a different colour scheme (e.g. with a coloured background) and translating English words into Arabic. **However**, interview also asked to change the calendar to the preferred one while UT asked to access health guide without requiring an internet connection. |
| **Data entry and navigation** | Problems raised among users affect their inability to see or find button due to unfamiliar terminology, too small button or buttons were in unclear location. They also suggested to enter BP, medication and stress once. |  | Participants expressed that the navigation was consistent when moving between screens and allowed them to enter information, respond to reminders, viewing information | Perceived app as easy to navigate because of being well-designed and easy to enter data and edit at any time, even retrospectively.  A difficulty was faced in entering ‘Tick’ feature or even undo it. They also encouraged to enter BP, medication and stress once. | **Discordant,** participants in interview and questionnaire found app easier to navigate and enter data than participants in the UT**.** Interestingly, users in UT were under controlled setting and they had not any previous training or practice. |
| **Theme 2: the usage of the app** |  |  |  |  |  |
| General satisfaction and use | People were generally satisfied to use this app and asked to continue using it; they found it easy to use and useful in increasing understanding and managing of their diseases. |  | People were generally satisfying with the app and preferred to keep using it again. | Participants were very satisfied and found it easily incorporated in their routines; they expressed to keep using it due to offering everything in one place and easily accessible location for their data anywhere at any time, good overview of BP level and it is easy to use | All three strands very **similar** but interview **expand further** **details** about the reasons why they are satisfied with this app. |
| App functionality use |  | The most commonly recorded measurement was BP: each user inputted this an average of 19.8 times. BP was recorded 416 times across all users, compared with medication and stress, which were recorded 234 and 246 times, respectively |  | Participants frequently recorded BP, followed by medication and stress and consider them as a positive feature | Both 2 strands **confirmed** that the most recorded data is BP, followed by stress and then medication |
|  |  | The most commonly accessed functionality was viewing the Logbook, with each user accessing this an average of 60.2 times. |  | Viewing data trends in a list or graph were the most common used and useful feature. | Both 2 strands **confirmed** that the most accessed feature was viewing data trends in a list, except viewing graphs. |
|  |  | The least accessed functionality was ‘Challenge Created’, with each user accessing this an average of 3.78 times, and a total of 72 challenges created across all users. |  | People reported that they used the app to set different types of challenges, the most common being entering BP and taking medication. Most participants reported setting challenges for other activities intended to increase exercise. A few participants also reported setting challenges to reduce stress. | They not confirmed or discordant. The most common set challenges were monitoring BP and taking medication compared with others. Most participants set challenges to increase exercise but few of them set to reduce stress. |
|  |  | The retention data for study participants was good  as user retention then gradually decreased, until day 30 when retention was 47.8%. More than ¾ of participants were in the meaningful sessions range of 30-60 seconds or longer. The average session duration was 1 minute and 35 seconds. The app was opened 21.43 times. |  |  | Nothing to integrate |
| External factors influencing use of the app |  |  |  | Range of external factors arisen either positively or negatively affected their use of the app; participants reported that Busy lifestyles and other health issues prevented some participants from undertaking and/or completing additional challenges (e.g. exercise). Family was also mentioned, as both a motivating and demotivating factor for using the app. | Nothing to integrate |
| **Theme3: Capacity to support self-management** |  |  |  |  |  |
| *A daily monitoring tool* |  |  | App perceived as an effective and helpful tool in supporting self-management by  performing self-assessment and tracking  activities. | App perceived as a powerful tool for facilitating self-management by offering a structured system more disciplined to manage their data, including BP, taking medication and emotions. Entering data was considered easier than conventional recording methods, or relying on memory. | Both 2 strands **confirmed** that the app perceived as a powerful tool for facilitating self-management by tracking different activities and data |
| *An informative tool* | Participants like the breadth of information and the provided information in addition to the feedback help to increase understanding and encourage to take action. |  | Participants like the provided health information in addition to the feedback that help them to know the progress of them. | Perceived as a powerful tool to receive feedback, and review educational resources about hypertension. The feedback functionalities were considered particularly valuable because they provide a clear picture of participants BP levels and see the relationship between their challenges completed and their BP level. some suggestion was commented like more detail and tailoring of BP feedback to their individual cases and too general, and would have preferred a breakdown of the specific tasks completed that week. | **Expansive**, further detail available from interview that **confirm** data and add more suggestions. |
| *A commitment tool* |  |  |  | Participants expressed that this app increased their commitment and encouraged them to add more self-management strategies to their routine. Some participants felt that additional information showing the daily activities they have to be prepared for would increase their completion of challenges, rather than relying on a notification alone. Participants appreciated the alerts advising them that certain activities for unsuited to their level of hypertension. | Nothing to integrate |
| *A communication tool* |  |  |  | Participants recognized that app improve patients’ communication and sharing of data at doctors’ visits. | Nothing to integrate |
