# Supplementary material for: Using a Commercially Available App for the Self-Management of Hypertension: Acceptance and Usability Study in Saudi Arabia
Source: JMIR Mhealth Uhealth. 2021 Feb 9;9(2):e24177. doi: 10.2196/24177 (PMC7902196; doi:10.2196/24177)
Supplement: Multimedia Appendix 2 [file mhealth_v9i2e24177_app2.docx]

A) User Interface Issues

| Subtheme | User Interface Issues | | | | | |
| --- | --- | --- | --- | --- | --- | --- |
| Categories | Unclear or misleading terminology | Unintuitive aspects of the design | | | Unclear Visual Presentation | |
| Usability issues | Difficulty setting reminders and reading health information | Difficulty checking completed tasks | Difficulty entering BP reading | Issues with inputting multiple data | Unclear colour-coding | Unclear colour contrast |
| Comments | Several participants found it difficult to find the correct menu to set reminders and read health information, due to unclear or misleading terminology. | Participants remarked that it should be easier to check how many tasks have been completed, e.g. by this being displayed when tasks are marked as complete, or being visible in the ‘Challenge’. | Half of the participants could not find the location to enter their BP mostly because they did not correctly interpret the ‘+’ sign. | Participants found the method of inputting data unintuitive, causing them to have to repeat the same procedures multiple times, rather than inputting all data (e.g., BP, medication, and stress) in one go^[[1]](#footnote-1)^. | The app presentation could be made clearer, by adding more text to some charts e.g., to explain the colour-coded classification of BP reading. | The colour of the buttons could be made more contrasting to accentuate some features. |
| Citation(s) | *“I do not believe the information is here…it cannot be found" (P3).* | *“The location of it [how many tasks have been completed] cannot be shown clearly - the box should appear directly when I tick for completing data.” (P9)* | *“I did not think that I can enter BP data via this + button” (P4)* | *“There is a problem in this app, as when I enter BP data... it backed me to the main menu … then I access ‘+’ to enter medication data and press save, then the app backed me again to the main menu [dashboard]… I have to access + again and enter stress then press save… It should allow for all data [BP, medication and stress] to be entered at the same time then save it]”. (P6)* | *She can find the data on which color, but she said “I cannot interpret whether it is normal or not; I see the colours, and don’t know what each colour means” (P9)* | *“It is better if it is made a bright color to clearly show that the other data needs to be added beside”. (P10)* |

B) App Accessibility

| Sub-Theme | App accessibility | | | |
| --- | --- | --- | --- | --- |
| Categories | Readability | | Additional barriers to use | |
| Usability Issues | Zoom feature | Font size | Language issues | Internet |
| Comments | Participants liked being able to zoom in on the health guide section as it made the text easier to read. | Some users stated that font size should be adaptable. | Participants experienced issues with the language of the app (certain words being untranslated) | Lack of internet access was a factor limiting some participants’ use of e.g. the health guide. |
|  | *“I understand the text, it is clear - and wow, I can zoom in” (P7)* | *“The font in the challenge menu is small and difficult to read~~.~~” (P10)* | *“I will select any medication name [because they cannot remember their medication name] as it is difficult to read it in English!” (P6).* | *“It needs internet to access. I cannot read it now until I get home.” (P6)* |

1. This issue only arose in the Arabic-language trial version, and was not present in the commercially available English-language version. [↑](#footnote-ref-1)
