# Supplementary figures and images for: Using a Commercially Available App for the Self-Management of Hypertension: Acceptance and Usability Study in Saudi Arabia
Source: JMIR Mhealth Uhealth. 2021 Feb 9;9(2):e24177. doi: 10.2196/24177 (PMC7902196; doi:10.2196/24177)

1. Self-monitoring of behaviour and feedback


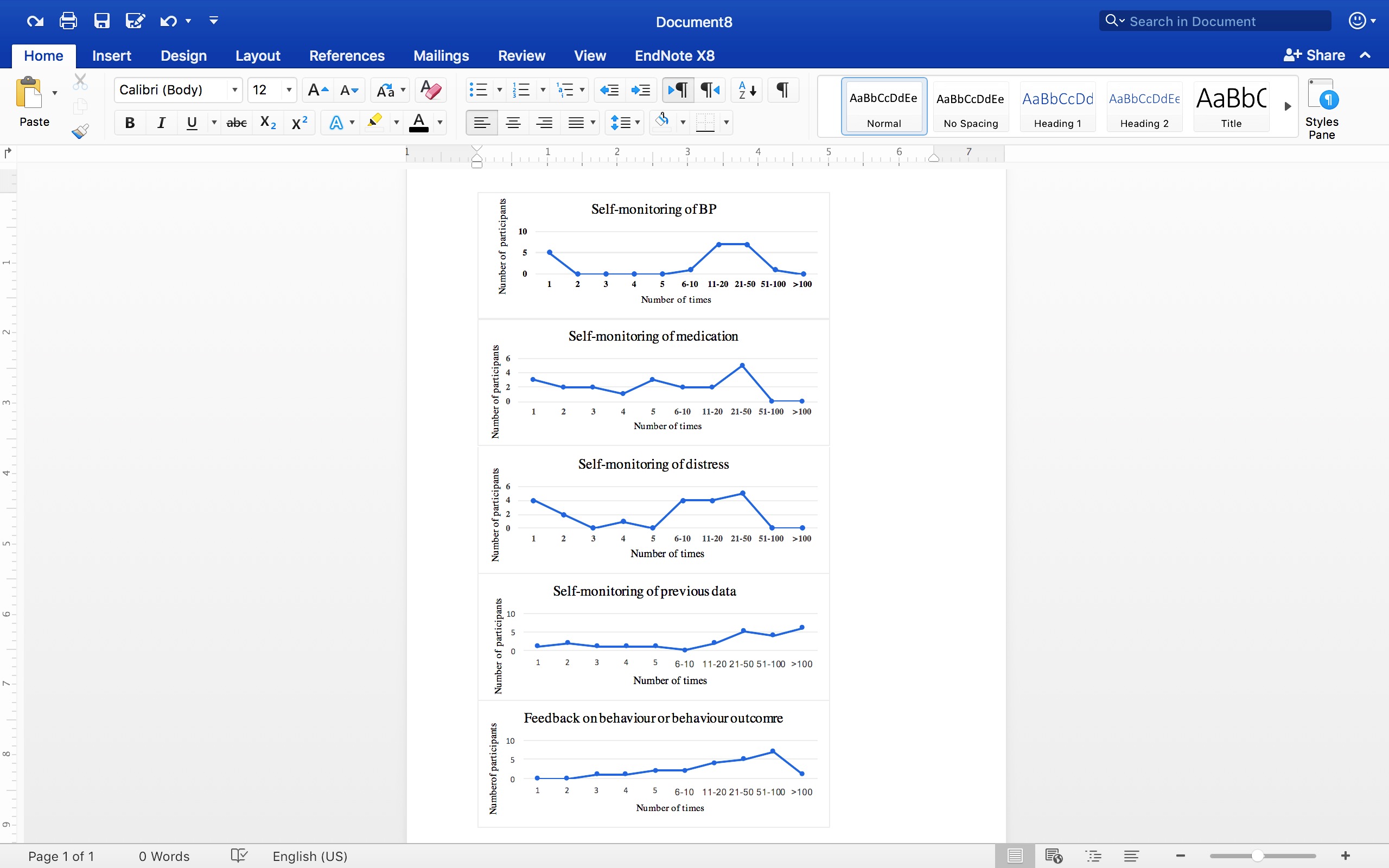


1. Goal Setting and Review


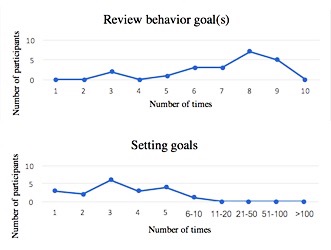

Supplement: Multimedia Appendix 3 [file mhealth_v9i2e24177_app3.docx]
