# Supplementary material for: Using a Commercially Available App for the Self-Management of Hypertension: Acceptance and Usability Study in Saudi Arabia
Source: JMIR Mhealth Uhealth. 2021 Feb 9;9(2):e24177. doi: 10.2196/24177 (PMC7902196; doi:10.2196/24177)
Supplement: Multimedia Appendix 4 [file mhealth_v9i2e24177_app4.docx]

| **Theme/Subtheme** | **Quotation** |
| --- | --- |
| **Usage of the app** |  |
| General satisfaction and use | *“First I felt using this app is effort and time consuming, but with over time, I see how it was benefits. I see all the data that I did and all the activities I completed and how it works to improve my BP. It is really interesting” (P10)*  *“I think this application is enjoyable as it did not consume my time or even require intensive effort.” (P12)*  *“Yes satisfied with it, as I said earlier, the level of my blood pressure is improved, it is accessible and convenient I can access it any time which is not like other conventional or manual tools. It supports me in tracking my health become more active with accurate picture of my data” (P7)*  *“I would like to say that anyone who wants to manage his blood pressure use this application because I found it is very useful” (P5)*  *“This app can be really used as an informative tool, storing my data, offering information etc.” (P6)*  *” I enjoy using it and become part of my daily life” (P6)*  *“I feel everything related to my hypertension is in one place” (P8)*  *” I was satisfied with it even it takes time, but make me more organized and the app is with me in my pocket at any time everywhere.” (P15).*  *“I felt using this app is effort and time consuming, but with over time, I see how it was benefit” (P10)*  *“I think using this app may require intensive efforts;” (P3)*  *“Of course, I will use it again and I will advise my friends about it because it provides a main location to input my data, which I can then access at any time to provide me with clear picture of my BP level.” (P10)*  *“After seeing its benefits, I definitely will continue using it without any doubt […] but please can I keep this version to use?” (P7)*  *“I love it because instead of holding actual logbook papers at each appointment where I was forgetting to take them [these papers] many times, it seems using this app was simpler and easier.” (P15)* |
| App Functionality use | *“I used each part of the app. The most common data I entered is BP. I had entered it every day … entering data is simple. I just monitor and the data is transmitted to the screen. I did not have to do anything more- that is why I love it” (P13)*  *“Using app is easy to enter my BP and it represents data in different, attractive ways, such as a list or graphs.” (P11)*  *“I used the app mainly to record my BP data, and I used it to record other data as much as possible such as medication and distress.” (P3)*  *“I just set goals for medication and BP monitor for every day. I did not add more because I am not interested in exercise or I am so busy when I get back home after work”*  *“When I notice my emotion level I was not happy After a while of time I set goal for reducing stress by do messages once a week” (P14)*  *“It requires additional information. […] I mostly eat Saudi foods that are not mentioned here” (P12)*  *“After days, I felt there is no any new information for me” (p17)*  *“It is not one function that is good. It is the combination of functionalities that is effective and valuable.” (P1)* |
| External factors influencing use of the app | *“My daughter helped me read some of the text and enter medication when I started using this app” (P5)*  *“My wife said that this app steal you from our life” (P13)*  *“I did not set other goals since walking is enough as I have pain in my joint that hurts me more after heavier exercise.” (P16)* |
| **Capacity to support self-management** |  |
| A daily monitoring tool | *“It changes the way that I manage my hypertension. Before, I just monitor my BP and ate medication, I did not record any task if it was completed. Using this app helps to follow up a system to achieve my goals.” (P13)*  *“When I use the BP monitor, my BP data directly moved to the app” (P2)*  *“I don’t keep track of my BP really, but I check it every two days or so and try to remember the reading […] [but with the app] it’s good to be able to enter data accurately and quickly and see the trends. It gives you a clearer picture.” (P7)*  *“I do not know why [my symptoms] are different – sometimes I have headaches, dizziness or I go to toilet many times a day. It would be good to add this feature.” (P13)*  *“The main thing I do is record my BP data and then check how my condition is and what the relationship is between my mood, taking medication and doing exercise, and my BP. If it is not improved, I try to read information to get more knowledge.” (P15)* |
| An informative tool | *“Sometimes I am confused about what the helpful treatment is - should I take more medication, drink fluid or ...; it’s very general- not very specific advice for my case.” (P8)*  *“I believe the information about how many tasks are completed would better if when I press it, it presents how many tasks from each goal that I set” (P15)* |
| A commitment tool | *“I know I’m going to do a lot more activities than I used to” (P2)*  *“As the reminder pushes me to do it. Even if sometimes I neglect it the additional reminders push me a lot.” (P11)*  *“The reminder pushes me but it would be better to show more [information] on the menu screen that today I have an activity for a walk.” (P8)*  *“I cannot add the challenge that I prefer, I just have to choose from what is here.” (P17)*  *“The negative part that there is no possibility to add/set any activity that I may need to” (P14)* |
| A communication tool | *“I have my iPhone and show the doctors how I did the activities during the previous days and what my BP level is*”. (P19)  *“Graphs are quickly and easily understood by both the doctor and me” (P6).*  “*Having this app helps me avoid repeating what I say each time I have new doctor at a clinic or in an emergency.” (P3)*  *“If the doctor can follow my recording, I will be more relaxed and also encouraged because he knows what I complete or not” (P15)* |
| **Usability of the app** |  |
| Overall usability | “*As the app is easy to use, my confidence to use it is high because it just needs me to select and click no more things”. (P14)*  “*I did not have practice using technology when I was young so I find it difficult at first but then with time the app becomes easier.” (P6)*  *“I found it easy at first because the brochure instructions helped me to understand how the app works. .... If I can’t do something at first I either read the instructions or ask my wife who has more experience of using apps in general. (P13)*  *“The button or menu can be found smoothly I did not face issue with them, date also entered and edited easily but there are some steps repeated at every time. For example, when I enter data (e.g., BP) it should be in one interface to enter all data. This lead me sometimes to enter only BP and forget to enter the other like medication.” (P4)*  *“The negative aspect is repeating the same procedures entering the BP, medication and stress data, it should be done once.” (P10)*  “*If you are unable to use your phone one day or forget to upload data, you can just add data later […] That for me is a big positive.” (P11*)  “*One time I accidentally ticked for walking although I had not achieved the task! So I feel it is better to allow me to edit or delete [a tick].*” (P9)  “*I have big fingers that make it hard to tick properly and sometimes my hands shake which also means I sometimes tick wrongly.*” *(*P13)  *“I think rather than having to go and tick after each task, it is easier after any reminder to show me a box asking me whether I have completed the task, then if I ‘tick’ it, it automatically records it.” (P4)* |
| App accessibility | *“I think this application is excellent and I am satisfied with it and I did not face any difficulty except font size.” (P11)*  *“Allowing us to customize the colors and size of fonts to meet our needs [would make the app more accessible]” (P5)*  *“I expect that the medicines need to be written in Arabic or an open option and the patient is the one who writes the name of the drug in Arabic” (P7)*  *“There were some issues that did not hinder my use but affect my use a little bit. For example, in Saudi we mostly use Islamic calendar in hospitals, schools and so on, and few use that English calendar, but in the app, I cannot set it to the Islamic calendar.” (P10)* |
